# HDL in sepsis – risk factor and therapeutic approach

**DOI:** 10.3389/fphar.2015.00244

**Published:** 2015-10-23

**Authors:** Emily E. Morin, Ling Guo, Anna Schwendeman, Xiang-An Li

**Affiliations:** ^1^Department of Pharmaceutical Sciences, College of Pharmacy, University of Michigan, Ann ArborMI, USA; ^2^Biointerfaces Institute, University of Michigan, Ann ArborMI, USA; ^3^Department of Pediatrics, Saha Cardiovascular Research Center, University of Kentucky College of Medicine, LexingtonKY, USA

**Keywords:** high-density lipoprotein (HDL), apolipoprotein A-1 (ApoA1), sepsis, lipopolysaccharide (LPS), sepsis therapy

## Abstract

High-density lipoprotein (HDL) is a key component of circulating blood and plays essential roles in regulation of vascular endothelial function and immunity. Clinical data demonstrate that HDL levels drop by 40–70% in septic patients, which is associated with a poor prognosis. Experimental studies using Apolipoprotein A-I (ApoAI) null mice showed that HDL deficient mice are susceptible to septic death, and overexpressing ApoAI in mice to increase HDL levels protects against septic death. These clinical and animal studies support our hypothesis that a decrease in HDL level is a risk factor for sepsis, and raising circulating HDL levels may provide an efficient therapy for sepsis. In this review, we discuss the roles of HDL in sepsis and summarize the efforts of using synthetic HDL as a potential therapy for sepsis.

## Introduction

Sepsis is a major health issue in the U. S., claiming over 215,000 lives and causing a financial burden exceeding $17 billion annually (Martin et al., 2003; Riedemann et al., 2003; Angus and van der Poll, 2013). The prognosis for sepsis remains grim, with a mortality rate exceeding 30%, due to poor understanding of the disease and a lack of efficient therapy (Sessler et al., 2004; Lagu et al., 2012; Dellinger et al., 2013).

**FIGURE 1:**
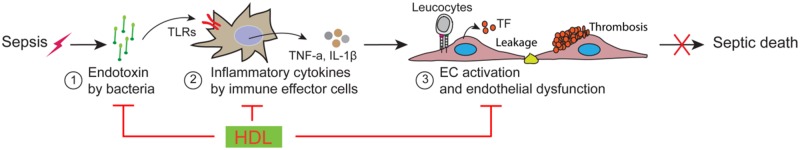
**Schematic model of vascular endothelial dysfunction in sepsis and high-density lipoprotein (HDL) protection against sepsis**.

A major contributor to sepsis mortality is the breakdown in the function of vascular endothelial cells (EC; Aird, 2003; Martin et al., 2003; Riedemann et al., 2003; Deanfield et al., 2007; Angus and van der Poll, 2013). As shown in **Figure 1**, this breakdown is caused by a cascade of inflammatory events-induced by infections, which includes three major factors/steps: (1) upon infections, bacteria release endotoxin; (2) endotoxin activates immune effector cells to produce inflammatory cytokines and chemokines; (3) inflammatory cytokines and chemokines activates EC, resulting in endothelial dysfunction manifested by vascular leakage, increased leukocyte adhesion, altered vascular tone and a shift in the hemostatic balance toward a pro-coagulant phenotype, which eventually leads to irreversible multi-organ failure and septic death (Aird, 2003; Martin et al., 2003; Riedemann et al., 2003; Deanfield et al., 2007; Angus and van der Poll, 2013). Thus, targeting endothelial dysfunction has been proposed as a potential sepsis therapy. A great challenge is that multiple factors/steps contribute to endothelial dysfunction in sepsis and simply targeting one of the regulatory factors/steps may have limited effect. Indeed, extensive efforts to block one or another component of the inflammatory or coagulation pathways have had little impact on patient survival (Fink and Warren, 2014).

We believe that targeting an endogenous factor with multi-protective effects against endothelial dysfunction may present a novel approach for sepsis therapy. Emerging evidence suggests that High-density lipoprotein (HDL) is likely such a candidate. In this review, we discuss the roles of HDL in sepsis and summarize the efforts of using synthetic HDL (sHDL) as a potential therapy for sepsis.

## Low HDL is a Risk Factor for Sepsis

High-density lipoproteins (HDL) are nanosized protein-lipid particles that circulate throughout the body as a major component of the blood (Navab et al., 2011; Hewing et al., 2012; Zhu and Parks, 2012). Of its physiological functions, HDL is most notably known for its role in cholesterol mobilization and inflammation. HDL, via reverse cholesterol transport, removes cholesterol from peripheral cells and transports it to the liver for excretion in the bile or transport to the adrenals, testes, or ovaries for hormone production (von Eckardstein et al., 2001; Lewis and Rader, 2005; Ohashi et al., 2005; Vickers and Remaley, 2014). In several clinical investigations, plasma HDL levels was shown to be inversely correlated with the occurrence of cardiovascular diseases (CVDs), and many patients with severe CVD have very low levels of circulating HDL (Nofer et al., 2002; Barter et al., 2004; Remaley et al., 2008). Thus, HDL has long been sought after as a possible therapy for the reversal of atherosclerosis and other CVDs. However, HDL is also an important player in inflammation and acute inflammatory disorders. Specifically, HDL has been shown to exert anti-inflammatory properties both *in vitro* and *in vivo* (Harris et al., 2002; Barter et al., 2004; Mineo et al., 2006; Argraves and Argraves, 2007; De Nardo et al., 2014; Lüscher et al., 2014) and levels of HDL in patients with inflammatory disorders, such as sepsis, have been proven to be prognostic of clinical outcomes (Gordon, 2004; Riwanto and Landmesser, 2013).

High-density lipoprotein is a strong indicator of both the onset and progression of sepsis. Clinically, HDL levels drop markedly in septic patients, and whether or not these levels rise or continue to fall is often foretelling of their chance of survival (van Leeuwen et al., 2003; Chien et al., 2005; Tsai et al., 2009). In a study of 63 patients, investigators found that those with plasma HDL concentrations exceeding 25 mg/dl at the time of intake had a 100% survival rate (Chien et al., 2005). Additionally, the investigators assessed the power of HDL to predict mortality rate by compartmentalizing patients into two groups: “low” (<20 mg/dl) and “high” (>20 mg/dl) HDL. Using these cutoff values, HDL had a sensitivity of 92%, a specificity of 80%, and an accuracy of 83% for predicting the overall 30-day mortality rate (Chien et al., 2005). The same was done for ApoA1 levels using a cutoff value of 100 mg/dL, however, ApoA1 showed an overall lower predictive value with an accuracy of 73% (Chien et al., 2005), aligning well with other studies showing that HDL, rather than lipid alone, is a better defender against septic shock (Levine et al., 1993).

Other investigations have also been carried out to determine whether low HDL causes septic death. Using ApoA1-null mice as a model for low circulating HDL, it was found that a deficiency in HDL leads to susceptibility to cecal ligation and puncture (CLP)-induced septic death, as well both decreased LPS neutralization and LPS clearance(Guo et al., 2013). Alternatively, increasing HDL levels by over expression of ApoAI improved the survival in both CLP and LPS-induced sepsis models (Li et al., 2008; Guo et al., 2013). Given these clinical and experimental data, we propose that low HDL is a risk factor for sepsis and that targeting HDL may provide an efficient and effective therapy for sepsis.

## Mechanisms of HDL Protection Against Sepsis

High-density lipoprotein is a potential multi-protective factor in sepsis. HDL has a broad spectrum of activity, including regulating both immunity and vascular EC functions (Singh et al., 2007; Navab et al., 2011). While most of the existing knowledge of HDL has been acquired in non-sepsis conditions, extensive evidence suggests that HDL likely plays pivotal protective roles in all the steps of endothelial dysfunction (**Figure 1**), including detoxification of endotoxin, suppression of inflammatory signaling in immune effector cells and inhibition of EC activation.

### Detoxification of Endotoxin

Bacterial infections are major causes of sepsis (Vincent et al., 2009). Upon infections, the Gram-negative bacteria release lipopolysaccharides (LPS) which bind to its receptor TLR4 to initiate a downstream signaling cascade. TLR4 binding leads to activation of proinflammatory genes to produce high levels of cytokines such as TNF-α and IL-6, resulting in cell damage (Raetz and Whitfield, 2002; Carmody and Chen, 2007). HDL is well known as a LPS neutralizer (Van Lenten et al., 1986; Flegel et al., 1989; Harris et al., 1990, 1993; Read et al., 1993; Eggesbo et al., 1996; Munford, 2005; Lee et al., 2007; Murch et al., 2007). Most LPS in circulation exist in HDL-bound form (Ulevitch et al., 1979, 1981), and HDL-LPS binding attenuates LPS-TLR4 inflammatory signaling in macrophages (Munford and Dietschy, 1985; Flegel et al., 1989; Baumberger et al., 1991; Emancipator et al., 1992). It is worth noting that simply neutralization of LPS may not provide efficient protection against sepsis which is shown by the failure of anti-LPS monoclonal antibodies in clinical trials (Cohen, 1999). We speculate that the failure of anti-LPS monoclonal antibodies in clinical trials could be attributed to: (i) the apparent inability of antibodies to block LPS-induced cytokine production in human monocytes *in vitro* (Warren et al., 1993; Cohen, 1999); and (ii) the fact that antibody partially sequester LPS, thus delaying rather than facilitating its clearance (Van Amersfoort et al., 2003). Recent studies including ours suggest that HDL acts together with its receptor, the scavenger receptor BI (SR-BI), to promote LPS clearance (Vishnyakova et al., 2003; Cai et al., 2008; Guo et al., 2009). *In vitro*, HDL promotes SR-BI-mediated LPS uptake by 4-fold in SR-BI-transfected HEK cells and by twofold in primary hepatocytes (Cai et al., 2008). *In vivo*, mice deficient in SR-BI or HDL display impaired LPS clearance in LPS or CLP animal models (Cai et al., 2008; Guo et al., 2009, 2013). These findings suggest that HDL neutralizes LPS and promotes LPS clearance via SR-BI-mediated LPS uptake, which presents a more efficient mechanism for LPS detoxification relative to neutralization by anti-LPS antibodies.

Lipoteichoic acid (LTA), released by Gram-positive bacteria, activates the TLR2/6 pathway to generate high levels of inflammatory cytokines, causing cell injury. Similar to LPS, most LTA are associated with HDL in circulation and this HDL-LTA binding neutralizes LTA (Grunfeld et al., 1999; Levels et al., 2001). Given the structural similarity between LPS and LTA, it is likely that HDL neutralizes LTA and promotes LTA clearance via SR-BI-mediated LTA uptake.

### Regulating Inflammatory Response in Macrophages

Macrophages are major immune effector cells responsible for inflammatory cytokine production in sepsis (Su, 2002). The inflammatory response in macrophages is required for fighting against infections. However, dysregulation of macrophages produces too many cytokines, leading to vascular endothelial dysfunction and organ injury in sepsis. A body of evidence indicates that HDL is a key modulator of inflammatory response in macrophages (Yvan-Charvet et al., 2008; Zhu et al., 2008; Suzuki et al., 2010; Mineo and Shaul, 2012; Zhu and Parks, 2012; De Nardo et al., 2014): (i) HDL promotes the eﬄux of free cholesterol from macrophages, resulting in suppression of LPS-induced inflammatory response in macrophages (Mendez et al., 2001; Puff et al., 2005); and ii) HDL upregulates the transcriptional regulator ATF3 which down regulates the expression of inflammatory molecules, resulting in suppression of the inflammatory response in sepsis (De Nardo et al., 2014).

### Regulating Endothelial Cell Function

Endothelial cells are activated by LPS and inflammatory cytokines (Aird, 2003; Deanfield et al., 2007; Shapiro et al., 2010). As discussed above, HDL can attenuate EC activation through its roles in promoting LPS detoxification and suppressing inflammatory cytokine production in macrophages. In addition, earlier studies demonstrated that HDL has a variety of activities that modulates EC functions, including: (i) inhibition of adhesion molecule expression stimulated by TNF-α, IL-1β or thrombin (Cockerill et al., 1995, 1999); (ii) activation of eNOS. NO generated by eNOS at small blood vessels is critical for promoting blood supply to small blood vessels and for inhibiting thrombosis in sepsis. Earlier studies including ours demonstrated that HDL activates eNOS to release NO in a SR-BI-dependent manner (Yuhanna et al., 2001; Li et al., 2002; Gong et al., 2003; Mineo et al., 2003); and (iii) prevention of endothelial thrombotic activation by promoting prostacyclin and Cox2 production and suppressing tissue factor and adhesion molecule expression (Ming et al., 2004; Viswambharan et al., 2004; Riwanto and Landmesser, 2013).

In conclusion, HDL likely plays critical roles in promoting LPS/LTA detoxification, suppressing inflammatory response in macrophages and inhibiting EC activation, which may present HDL a multi-protective factor against endothelial dysfunction in sepsis.

## Synthetic HDL is a Potential Effective Therapy for Sepsis

Reconstituted or sHDL made from ApoAI protein or ApoAI mimetic peptide presents a new strategy for promoting the biological activity of HDL (Krause and Remaley, 2013). Experimental and clinical investigations, including phase 2 clinical trials for treatment of CVD, have shown that infusion of sHDL raises circulating HDL levels, improves endothelial function and reduces platelet aggregation (Patel et al., 2009; Krause and Remaley, 2013), and with HDL levels at the time of hospitalization being positively correlated with increased survival rates among septic patients (Barlage et al., 2009), it is reasonable that HDL replacement therapy has been a well sought-after area of sepsis research. Not only does HDL confer cardio-protection via maintaining endothelial barrier integrity and reverse cholesterol transport, it is also able to combat inflammation and oxidization, as shown both *in vitro* and *in vivo* (Pajkrt et al., 1996). Several studies have been carried out in order to investigate the protective ability of administered HDL against endotoxemia, a few of which are discussed below, and can be found summarized in **Table 1**.

**Table 1:** Experimental high-density lipoprotein (HDL) therapies in animals models of sepsis and their outcomes.

HDL	Dose and administration	Sepsis model	Main findings	Reference
18A:Egg PC (1:2 wt/wt) sHDL	80 mg/kg; prophylactic tail vein IV infusion	Swiss Webster mice; LPS (*salmonella*) 10 mg/kg; IP injection; within 15 min of HDL	Three–fourfold increase in 48-h survival rate vs control (*p* < 0.05)	Levine et al., 1993
L-4F Peptide	25 mg/kg, IP, concurrently with lipopolysaccharide (LPS)	Sprague-Dawley rats; 10 mg/kg LPS; IP injection	Reduction in VCAM-1 expression in excised aortae	Gupta et al., 2005
4F Peptide	10 mg/kg; IP injection post-LPS challenge	Sprague-Dawley rats; LPS 10 mg/kg or 30 mg/kg; IP injection	10 mg/kg LPS: 4F slowed LPS plasma clearance; reduced hypotension at 6 h; 30 mg/kg LPS: 4F increased plasma HDL levels; increased 24-h survival	Dai et al., 2010
4F Peptide	10 mg/kg, IP, 6 h post-CLP	Sprague-Dawley rats; CLP	Reduced IL-6; restored CO, right atrial pressure, and plasma volume; improved 2-day survival rate; reversed sepsis-induced changes in lipoprotein profile	Zhang et al., 2009
4F Peptide	10 mg/kg; IP injection 6 h post-cecal ligation and puncture (CLP)	Wistar Rats; CLP	Restored renal, hepatic, and cardiac functions; reduced renal tubule damage; restored expression levels of Slit2, Robo4, and eNOS; increased plasma HDL; improved 4-day survival; no change in MAP	Moreira et al., 2014
D-4F Peptide	20 μg daily for 9 days; IP injection	C57BL/6J mice; nasally innoculated with 10^5^ PFU influenza virus A/WSN/33	Prevented lymphoid hyperplasia; increased PON activity; prevented drop in core body temperature; suppressed plasma IL-6 levels; increased plasma HDL and inhibited lipoprotein alteration; reduced viral titers by >50% at all time points	Van Lenten et al., 2002
ApoA1	10 mg/kg; IP injection 1 h post-LPS challenge	Wistar rats; LPS 1 mg/kg (TNF analysis) or 5 mg/kg (survival study); IP injection	Reduced plasma TNF-α levels in rats given 1 mg/kg LPS; increased 5-day survival rate from 0 to 90% in rats given 5 mg/kg LPS	Imai et al., 2003
ApoA1, human plasma purified	100 mg/kg; IV infusion post-LPS challenge	Balb/c mice; LPS 5 mg/kg; IP injection	Increased both survival rate and average survival time over 3 days	Yan et al., 2006
ApoA1 Milano ApoA1:Soy PC (1:3.35 mol/mol) rHDL	40 mg/kg; prophylactic IV injection	Wistar rats; 400 EU/kg Gram-negative bacterial endotoxin; IV injection	Increase in HDL-C; improved renal and hepatic function; inhibition of cytokines TNF-α, IL-1β, IL-6; reduced expression of ICAM-1	Zhang et al., 2015
ApoA1:Soy PC (1:200 mol/mol) rHDL	75 mg/kg ApoA1; prophylactic continuous IV infusion over 25 min	NZW rabbits; LPS 25 μg/kg continuous IV infusion over 6 h; start 20 min post-rHDL treatment	Complete inhibition of TNF-α; prevented LPS-induced hypotension; reduced metabolic acidosis; no significant effect on serum LPS levels	Hubsch et al., 1993
ApoA1:Egg PC (1:2 w/w) rHDL	500 mg/kg ApoA1; IV infusion at 0.1 g/kg/hr; split into three doses: 0.3, 0.1, and 0.1 g/kg administered at 0.5, 8, and 16 h post-infection, respectively	2-year old Beagles surgically implanted with E. coli-infected fibrin clot	Reduced plasma endotoxin levels; decreased plasma TNF-α; decreased liver function; decreased 2-day survival and average survival time	Quezado et al., 1995
CSL-111 ApoA1:Soy PC (1:150 mol/mol) rHDL	75 mg/kg ApoA1; (a) Prophylactic IV infusion over 40 min (b) Treatment by IV infusion over 20 min, 1 h post-bacterial challenge	NZW Rabbits (1) Gram (-) Sepsis: 4 × 10^9^ CFU/kg E. coli; IV infusion over 2 h (2) Gram (+) Sepsis: 2 × 10^9^ CFU/kg; IV infusion over 2 h	(1 a) Prophylactic rHDL: reduced plasma LPS and TNF-α; reduction in metabolic acidosis; no effect on hypotension or blood bacterial levels (1 b) rHDL Treatment: reduction in LPS after 4 h; reduced metabolic acidosis and creatinine; no effect on blood bacterial counts or TNF-α; no effect on hypotension (2) No effect in Gram (+) sepsis	Hubsch et al., 1995
CSL-111 ApoA1:Soy PC (1:150 mol/mol) rHDL	25 or 50 mg/kg ApoA1; Prophylactic IV infusion over 40 min	NZW rabbits; LPS 10 μg/kg; continuous IV infusion over 2 h; start 15 min post-rHDL completion	Reduced TNF-α levels and increased TNF-α clearance for both rHDL doses; rHDL 50 mg/kg reduced hypotension at *t* = 3–4 h; no effect on plasma LPS levels; no effect on blood leukocyte count	Casas et al., 1995
CSL-111 ApoA1:Soy PC (1:150 mol/mol) rHDL	40 mg/kg; prophylactic IV infusion over 4 h	Healthy male volunteers (20–28 years); Endotoxin 4 ng/kg IV bolus; given 3.5 h post-rHDL start	Elevated HDL levels; reduced endotoxin-induced clinical symptoms, i.e., chills, myalgia, backache, nausea, and vomiting; reduced plasma cytokine levels of TNF-α, IL-6, and IL-8; inhibited early leukopenia, monopenia, and neutropenia; reduced monocyte CD14 expression	Pajkrt et al., 1996

### ApoA1

Since ApoA1 is the main protein component of HDL, it makes sense that administering additional ApoA1 protein can increase circulating levels of HDL, avoiding the need to reconstitute with lipid, which adds an additional level of complexity. Administration of naked ApoA1 purified from human plasma has shown to have some beneficial effects in both rat and mouse LPS-induced endotoxemia models. When administered at 10 mg/kg (IP) 1 h post-infection, ApoA1 increased the 5-day survival rate from 0 to 90% in rats given 5 mg/kg LPS (Imai et al., 2003). In a similar model in mice, ApoA1 dosed 1 h post-infection via intravenous (IV) infusion at 100 mg/kg increased both 3-day survival rate and overall survival time versus saline-treated controls (Yan et al., 2006). Additionally, it was found that ApoA1 overexpressing mice were more resistant to infection than those with normal or decreased levels of circulating ApoA1 (Li et al., 2008).

### ApoA1 Milano

ApoA1 Milano is a naturally occurring variant of ApoA1 found in a select subset of individuals. Those carrying this mutation, despite having markedly lower levels of circulating HDL, have a much lower risk of developing CVD than their wild-type counterparts (Nissen et al., 2003; Nicholls et al., 2011). It was since developed as an reconstituted HDL (rHDL) therapy by Esperion Therapeutics where it entered a Phase I trial (Nicholls et al., 2011), however, after being licensed to Pfizer it forewent further clinical trials. While a majority of research around ApoA1 Milano is focused around CVD, rHDL using this variant protein (rHDL_M_) has also been shown to be efficacious against inflammation (Zhang et al., 2015). In a Gram-negative bacterial rat model, rHDL_M_ given prophylactically at 40 mg/kg was effective in suppressing pro-inflammatory cytokines TNF-α, IL-1β, and IL-6 (Zhang et al., 2015). Additionally, rats dosed with rHDL_M_ displayed increased renal and hepatic function as well as a decrease in cardiac tissue damage when compared to saline-treated controls (Zhang et al., 2015).

### CSL-111

CSL-111 is a rHDL originally produced by CSL Behring for the intention of treating atherosclerosis, making it through Phase II Clinical Trial before being superseded by CSL-112, CSL Behring’s current investigational rHDL therapeutic (Tardif et al., 2007). Made from purified human ApoA1 and soybean phosphatidylcholine (PC) at a molar ratio of 1:2 protein to lipid, CSL-111 has repeatedly shown efficacy in reducing the burden of LPS-induced endotoxemia both *in vitro* and *in vivo* in rabbit and human models (Casas et al., 1995; Hubsch et al., 1995; Pajkrt et al., 1996). In doses ranging from 25 to 75 mg/kg body weight, CSL-111 was able to suppress production of pro-inflammatory cytokines TNF-α, IL-6, and IL-8 (Hubsch et al., 1995; Pajkrt et al., 1996), inhibit sepsis-induced hypotension (Casas et al., 1995; Pajkrt et al., 1996), and markedly decrease the severity of clinical symptoms when administered prophylactically (Pajkrt et al., 1996). Although CSL-111 showed less promise when administered as treatment 1 h post-bacterial challenge, it was still able to reduce the degree metabolic acidosis and improve kidney function over saline controls in rabbit models (Hubsch et al., 1995).

### HDL Mimetic Peptides

While HDL therapy may be a feasible solution in the treatment of sepsis, the ability to produce therapeutic quantities of HDL is both a rate- and cost-limiting process in its development. For this reason, the use of ApoA1 mimetic peptides has gained increasing popularity, as they are a cheaper and easier way to make HDL-like particles.

Several mimetic peptides have been synthesized and studied to date, all of which are structurally similar to the amphipathic alpha-helices of native ApoA1 (Navab et al., 2005). Both *in vitro* and *in vivo* these peptides are able to bind phospholipids and associate with native HDL particles (Navab et al., 2005, 2011). Functionally, these mimetic peptides are able to reproduce the role of native HDL in their ability to eﬄux cholesterol (Sethi et al., 2008; Amar et al., 2010), interact with HDL receptors, i.e., ABCA1, ABCG1, and SR-B1 (Sethi et al., 2008), as well as interact with HDL-associated enzymes LCAT and PON (Van Lenten et al., 2002; Chen et al., 2009; Navab et al., 2011), and most relevant to sepsis, the ability to bind and neutralize LPS (Gupta et al., 2005; Remaley et al., 2008).

### 18A Peptide

One of the first HDL mimetic peptides to be investigated is the 18 amino acid peptide, 18A (DWLKAFYDKVAEKLKEAF). This peptide closely mimics the amphipathic alpha-helical structure of ApoA1, rendering it suitable for sHDL studies. *In vivo*, prophylactic infusion of 18A sHDL composed of 18A:Egg PC at a 1:2 weight ratio increased the survival rate three–fourfold over saline-treated controls in Swiss Webster mice infected with LPS from salmonella (Levine et al., 1993). Since, 18A peptide has been modified to create 4F peptide, which is discussed below.

### 4F Peptide

4F is an 18 amino acid peptide (DWFKAFYDKVAEKFKEAF) derived from the sequence of 18A, replacing two lysine residues with phenylalanine (Navab et al., 2005). 4F peptide has been the focus of several studies, and is synthesized using either L- or D-amino acids (L-4F and D-4F, respectively). D-4F is of particular interest to researchers because it can be delivered orally due its resistance to enzymatic degradation (Navab et al., 2005). In a mouse influenza model, D-4F was able to suppress IL-6 production, prevent lymphoid hyperplasia, maintain normal core body temperatures, and reduce viral titers by >50% over the entire course of study when administered intraperitoneally at doses of 20 μg daily (Van Lenten et al., 2002). L-4F has been more widely studied in the context of sepsis, and has been shown to be efficacious in both rat CLP and LPS-induced endotoxemia models at doses as low as 10 mg/kg body weight (Gupta et al., 2005; Zhang et al., 2009; Dai et al., 2010; Moreira et al., 2014). In such studies, L-4F administered by IP injection was shown to block production of cytokines TNF-α and IL-6, reverse sepsis-induced hypotension, prevent organ damage, and restore renal, hepatic, and cardiac function, and increase both survival rate and average survival time in comparison to saline-treated controls (Gupta et al., 2005; Zhang et al., 2009; Dai et al., 2010; Moreira et al., 2014). Most notably, L-4F was given after infection, rather than prophylactically, representing a more clinically relevant application and meriting its further investigation as a potential therapeutic.

### Prospectives in sHDL Sepsis Therapy

While previous studies have shown encouraging results, the earlier generation of sHDL and naked ApoAI mimetic peptides suffer from poor purity, short circulation times, contaminations, and toxicity (Quezado et al., 1995; DiPiro et al., 1996; Tardif et al., 2007; Zhang et al., 2009). It is worth noting that the current literature describing sHDL largely focuses on its protein/peptide composition and on its capacity in mediating cholesterol eﬄux. Considering that sHDL likely plays multi-protective roles in sepsis and the lipid components significantly alter the properties of sHDL, further efforts are required to understand these protective roles and tailor sHDL composition for increased efficacy in sepsis. Extensive efforts have been made to understand the roles of HDL/sHDL in CVD and other chronic inflammatory diseases, and these studies have profoundly improved our understanding about HDL/sHDL. We may take these advantages to further determine the roles of HDL/sHDL in the context of sepsis and develop the new generation of sHDL for sepsis therapy.

## Conflict of Interest Statement

The authors declare that the research was conducted in the absence of any commercial or financial relationships that could be construed as a potential conflict of interest.
